# Environmental influences on energy balance-related behaviors: A dual-process view

**DOI:** 10.1186/1479-5868-3-9

**Published:** 2006-05-15

**Authors:** Stef PJ Kremers, Gert-Jan de Bruijn, Tommy LS Visscher, Willem van Mechelen, Nanne K de Vries, Johannes Brug

**Affiliations:** 1 Department of Health Education and Health Promotion, University of Maastricht, P.O. Box 616, 6200 MD Maastricht, The Netherlands; 2 Department of Nutrition and Health, Vrije Universiteit, Amsterdam, The Netherlands; 3 National Institute of Public health and the Environment, Bilthoven, The Netherlands; 4 Department of Public and Occupational Health and Institute for Research in Extramural Medicine, VU University Medical Centre, Amsterdam, The Netherlands; 5 Department of Public Health, Erasmus MC, Rotterdam, The Netherlands

## Abstract

**Background:**

Studies on the impact of the 'obesogenic' environment have often used non-theoretical approaches. In this journal's debate and in other papers authors have argued the necessity of formulating conceptual models for differentiating the causal role of environmental influences on behavior.

**Discussion:**

The present paper aims to contribute to the debate by presenting a dual-process view on the environment – behavior relationship. This view is conceptualized in the EnRG framework (Environmental Research framework for weight Gain prevention). In the framework, behavior is postulated to be the result of a simultaneous influence of conscious and unconscious processes. Environmental influences are hypothesized to influence behavior both indirectly and directly. The indirect causal mechanism reflects the mediating role of behavior-specific cognitions in the influence of the environment on behavior. A direct influence reflects the automatic, unconscious, influence of the environment on behavior. Specific personal and behavioral factors are postulated to moderate the causal path (i.e., inducing either the automatic or the cognitively mediated environment – behavior relation). In addition, the EnRG framework applies an energy balance-approach, stimulating the integrated study of determinants of diet and physical activity.

**Conclusion:**

The application of a dual-process view may guide research towards causal mechanisms linking specific environmental features with energy balance-related behaviors in distinct populations. The present paper is hoped to contribute to the evolution of a paradigm that may help to disentangle the role of 'obesogenic' environmental factors.

## Background

Acknowledging the importance of discussions regarding the usefulness of current theories in the field of behavioral nutrition and physical activity, the IJBNPA has encouraged a debate on this issue [1]. Jeffery [2] stated that current popular health behavior theories overestimate the role of cognitive determinants, and that models are needed that address relationships between the environment and behavior. In line with this call for an increased focus on the role of environmental factors, Brug and colleagues [3] proposed the development and application of behavior change theories that focus on how to promote action rather than mere motivation. Rothman [4] focused on the important role of intervention research in theory development and he argued that greater attention should be paid to the causal processes invoked by potential moderators of intervention effects. The present paper aims to contribute to the debate by integrating these calls and those of others (e.g., [5-7]) into one conceptual framework. A dual-process model is outlined that can be used to gain insight into the causal mechanisms that underlie the relationship between environmental influences and behavior. This paper will specifically focus on behaviors that may positively or negatively influence the energy balance. We will refer to these behaviors as 'energy balance-related behaviors' (EBRBs).

### Toward a conceptual framework of determinants of EBRBs

The past decade, the importance of the 'obesogenic environment' has been highlighted [5,8,9]. However, recent reviews [10-12] have shown a lack of consistent results regarding the impact of environmental factors on EBRBs. A meta-analysis [13] confirmed the ambivalence in current empirical evidence. Based on sixteen studies, no single 'crude' environmental factor could be identified as consistently related to physical activity.

The evidence regarding environmental determinants of EBRBs collected to date has often been the result of non-theoretical approaches [7], which do not provide any knowledge on causal relationships between the identified associates of EBRB. Particularly, a lack of conceptual models for differentiating the causal role of environmental influences on behavior has been identified [14]. As a result, Owen and colleagues [7] urged researchers to go beyond looking at environmental attributes on their own and to systematically study the most relevant environmental influences of physical activity behaviors. Using the application of knowledge mapping techniques, a panel of experts from diverse professional fields concluded that "research is needed to document the extent of environmental influences [on physical activity and dietary behaviors] and *how *they affect different individuals" ([5] p. S35, italics added). In order to do so, more conceptually refined models of how environments might affect behavior are necessary, such as whether they affect behavior directly or through mediating variables [6].

### Dual-process view on environment-behavior relationship

Dual-process models in social psychology, such as the Elaboration Likelihood Model [15] and the MODE model [16] have conceived information processing as happening along a continuum. The anchors of this continuum reflect the 'duality' invoked by these models [17]. On the one hand, people can utilize no cognitive effort, elaboration, or capacity in engaging in a particular act. Behaviour can be the result of direct 'automatic' responses to environmental cues [18]. On the other hand, people can spend a great deal of time, effort and mental energy in systematically building beliefs and decisions. We postulate that the application of the dual-process view in the study of determinants of EBRB will help to gain insight into the circumstances under which EBRB is a conscious action or an action that is spontaneously or automatically performed under direct environmental control. Since it is inefficient to change cognitive factors regarding specific actions when these actions are unmediated by cognitions, such insights are highly relevant in order to inform intervention development.

**Figure 1:**
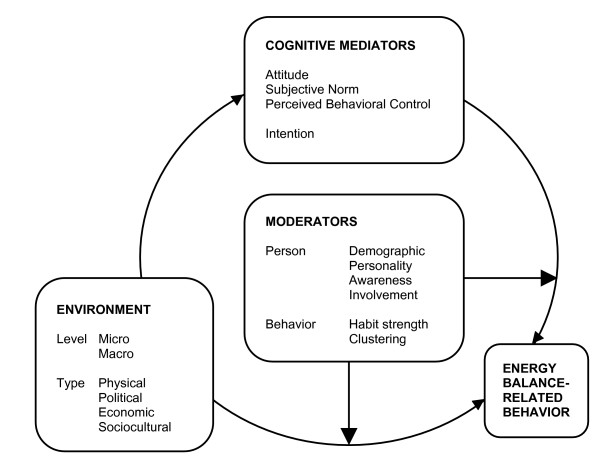
Environmental Research framework for weight Gain prevention (EnRG).

In the following sections, the Environmental Research framework for weight Gain prevention (EnRG; Figure 1) is proposed as a dual-process model that can be used to gain insight into the most important determinants of EBRBs as well as into the causal mechanisms that underlie these behaviors. First the conceptualization of energy balance-related behavior and the 'obesogenic' environment is shortly outlined. Then, mediated and unmediated environment – behavior processes will be discussed, followed by a description of specific potential moderators of the environment – behavior relationship.

### Energy balance-related behavior

Weight gain, overweight and obesity have been associated with various dietary behaviors such as a diet high in fat or carbohydrates and low in fiber, frequent snacking and frequent consumption of soft drinks containing sugar [19]. Physical activity is of similar complexity, consisting of a large variety of behaviors such as transport-related behavior, work-related activities, leisure time activities, and sport participation [20]. Low levels of physical activity as a part of daily routines have been particularly identified as weight gain-related risk behavior [21]. Note that it is impossible to focus on any single factor as a universal causal factor in obesity. It is the co-existence and interaction of these specific nutrition and physical activity behaviors that determines whether or not positive energy balance and weight gain are experienced [22]. Moreover, specific behavioral determinants of a positive energy balance will differ for different target groups (e.g. children; see [23]), emphasizing the importance of thorough epidemiological investigations. Such studies of behavioral causes of weight gain should form the basis for investigations of determinants of these particular behaviors ([6]; see [24] for methodological considerations regarding this issue).

### Environment

Various conceptualizations of the environment have been proposed, most of them specifically applied to either physical activity or diet. Three frameworks have specifically addressed potential environmental determinants of EBRBs. These models are the ANGELO framework (ANalysis Grid for Environments Linked to Obesity; [9]), the Framework for Determinants of Physical Activity and Eating Behavior [5] and the framework recently developed by the Committee on Prevention of Obesity in Children and Youth [25]. These models do not make inferences on the causal mechanisms that link the environment to behavior, but they are useful in conceptualizing potential determinants.

Swinburn et al. [9] have tried to divide the variety in types of 'obesogenic' factors into four distinct types of influence: physical (what is available), economic (what are the costs), political (what are the rules), and sociocultural (what is the social and cultural background). In addition, two levels of influence are distinguished: micro-environmental settings and macro-environmental sectors. Individuals interact with the environment in multiple micro-environmental settings, including schools, workplaces, homes and neighborhoods, which are, in turn, influenced by broader macro-environments, including health systems, governments and the food industry. When types and level of environment are crossed, it forms a grid that comprises four types of environment on one axis and two sizes of environment on the other. The application of the ANGELO framework has proven useful to categorize determinants of physical activity and dietary behavior as well as current efforts in the field of environmental interventions with respect to these behaviors [26]. With regard to the conceptualization of environmental factors, the EnRG framework has adopted the ANGELO grid. In order to guide the formulation of specific hypotheses regarding the working mechanisms of potentially relevant environmental factors, the EnRG framework explicitly postulates environmental factors to interact with each other (see Discussion section for an elaboration of this issue).

### Mediated environmental influences

Health education research and related behavior change studies have mostly been focused on proximal, cognitive, determinants of health-related behaviors [27]. A number of different theories and models have been proposed to study health-related behaviors, such as the Health Belief Model [28], Protection Motivation Theory [29], the Transtheoretical Model [30] and the Theory of Planned Behavior [31]. Based on a review of current health behavioral change models, Baranowski and colleagues [6] concluded that the Theory of Planned Behavior (TPB) is the most useful model for investigating cognitive determinants in the field of weight gain prevention. In the TPB, attitude, subjective norm and perceived behavioral control are the central cognitive factors. These factors are believed to influence the behavioral intention, which is assumed to be the primary determinant of behavior. Indeed, there is a large body of evidence showing that these cognitive factors are indeed associated with intentions and behavior for many health behaviors, including EBRBs (see [32,33] for reviews).

Some environments will mainly impact EBRB through its influence on attitude, other environments will mainly impact subjective norms and others will influence behavior through perceived behavioral control. For example, poor accessibility of health foods may reduce self-efficacy expectations towards healthy eating, high prices of health foods may have a negative impact on attitudes related to healthy eating, and poor availability of exercise opportunities may result in perceived norms that are negative towards physical activity. Changes in these cognitive factors are theorized to lead to changes in intention and subsequent behavior [31]. Empirical evidence exists regarding the mediating role of cognitive factors such as attitude or perceived behavioral control of environmental factors on physical activity behavior [34,35] and dietary behavior [36,37]. A review of physical activity intervention studies that have incorporated cognitive mediators in their research design provides some evidence for the mediating role of self-efficacy in intervention effects [38]. The mediated route from environment to behavior will hold in various instances, persons and behaviors studied. However, the cognitively mediated route does not always provide a sufficient account for variations in behavior. Studies that have combined environmental factors and TPB variables have shown that environmental factors have explained additional variance in behavior, over and above the cognitive factors (e.g., [35,36,38,39]). Findings of this kind are sometimes explained in terms of methodological flaws [40], but we postulate unmediated environmental effects to be important explanatory mechanisms in the field of EBRB.

### Unmediated environmental influences

The notion of least effort or limited capacity [16] has been suggested to result in 'mindless', automatic, processes. Since cognitive capacity is bounded and limited, automatic mental processes free one's conscious capacity from tasks in which they are no longer needed [17]. In this view, it would be impossible to function effectively if individuals have to deal with every aspect in life, from perceptual comprehension of the environment to choosing and guiding every action and response to the environment, in a conscious, controlled, and aware fashion [17].

The research field of automaticity has been growing exponentially over the past few decades [17]. Automatic processes can include attitude activation, automatic evaluation and emotion, unconscious behavioral mimicry, automatic trait and stereotype activation, and unconscious goal pursuit [41]. Usually, individuals are unaware of the automatic environment – behavior link, but lack of awareness is a sufficient but not necessary condition for automaticity. There are four components of automaticity: lack of awareness, lack of control, efficiency and lack of intent [42], but not all four need to be present for a process to be automatic (and rarely are; [41]).

Since the line of research presented above has not been specifically tested in the EBRB domain, empirical evidence is limited. However, experimental studies, mostly executed in laboratory settings, have provided indications on the existence of unmediated environment – behavior links in EBRB. Here, we will present some of these studies in order to show a variety of types of potential automatic environment – behavior processes: automatically activated goal-directed behavior, behavioral mimicry, implementation intentions, and body feedback.

Aarts & Dijksterhuis [43] showed that when consistent choices are made to reach certain goals, the action is automatically activated upon the activation of the goal. In a student population, it was found that the presentation of the location 'university' automatically activated the travel mode 'bicycle use'. This 'university-bicycle link' was found to be difficult to suppress or control, indicating that active transport can become automatically associated with travel goals and thus bypass behavior-specific cognitive factors [43]. Recent research has shown that consumer-related images (e.g. brands and their logos) can serve as environmental triggers of unconscious goals. Subliminal exposure to consumer brand logos has been found to influence consumers' actual behavior independently of previous behavior or existing brand-specific attitudes [41].

Investigations in the field of unconscious behavioral mimicry have shown that individuals can mimic gestures, postures and mannerisms, as well as consumption behavior (e.g., [44]). Ferraro et al. (see [41]) showed that participants in an experiment unconsciously mimicked a confederate's eating behavior (i.e. consumption of a specific type of crackers). Importantly, this study showed that, when asked to explain their dietary behavior, none of the participants mentioned the confederate in general, their eating behavior or the mimicry thereof in particular. Instead, they attributed their behavior to pre-existing evaluations and beliefs regarding the snack.

Studies with respect to 'implementation intentions' [45,46] have provided supporting evidence regarding automatic environment – behavior relations. Implementation intentions are concrete plans of action that specify when, where and which actions should be taken to achieve an intended goal. Although this is a highly conscious action, the working mechanism of implementation intentions is postulated to involve an automatic behavioral response to specific environmental cues. Actions have gained a degree of automaticity by being under control of relevant situational cues [47]. In the field of EBRB, Verplanken & Faes [48] demonstrated that implementation intentions to eat healthy were effective in establishing a more healthy diet, additive to the prediction of healthiness of eating by behavioral intentions. Armitage [49] showed in a controlled trial that fat intake, saturated fat intake, and the proportion of energy derived from fat decreased significantly in a group that formed an implementation intention regarding fat consumption but not in a control group. Notably, the difference between the experimental and control group could not be explained by differences in motivation.

Environments influencing body position or emotion may affect EBRB without individuals being aware of it. For example, Förster [50] showed that subjects who were asked to extend their arm (giving rise to bodily feedback associated with avoiding negative stimuli) consumed less cookies while watching a TV program for about 25 minutes than subjects who were asked to perform arm flexion (which gives rise to bodily feedback that signals a benign environment). Participants were not aware of the effect, and quality of taste, mood or feelings of pleasantness of the body position did not mediate the effect. Berridge and Winkielman [51] showed that thirsty participants exposed to subliminally presented happy faces (reflecting positive affective social environmental influences) consumed about 50% more of a fruit-flavored drink than thirsty participants who were exposed to subliminally presented neutral faces. Again, these individuals were unaware of the reaction at the moment it was caused.

### Moderators

In the EnRG framework, environmental influences are hypothesized to influence EBRB both indirectly and directly. The indirect causal mechanism reflects the mediating role of behavior-specific cognitions in the influence of the environment on behavior. The direct influence reflects the automatic, unconscious, influence of the environment on behavior. The postulation of behavior to be the result of a simultaneous influence of conscious and non-conscious processes represents the dual-process view. Various specific factors are postulated to moderate the causal path (i.e., inducing either the automatic or the cognitively mediated environment – behavior relation).

As argued in previous sections, EBRBs are likely to be complex. Consequently, the presence of interaction terms seems to be likely, and, as Baranowski et al. [6] argued, these terms should be sought. To the extent that one wishes to increase insights into causal pathways beyond the limits of models such as the TPB, it is useful to explore the more complex interactions involved in the mechanisms underlying the behaviors in question [52]. In the EnRG framework, the level of cognitive mediation of the environmental influences on behavior is postulated to differ along the lines of person- and behavior-related factors. Note that these factors can influence the level of cognitive mediation by either moderating the environment – cognition relation (i.e., the extent to which the environment induces behavior-specific cognitions) or the cognition – behavior relation (i.e., the extent to which the cognitions lead to actual engagement in the behavior). Six types of factors are specifically proposed: demographic factors, personality, awareness, involvement, habit strength and engagement in clustered behavior. The rationale for including these potential moderators in the framework is elaborated below.

#### Demographic factors

It has been suggested that environmental factors may have differential effects on various demographic sub-groups of the population [13,53]. Although few studies have systematically explored this moderating role of demographic factors in the environment – behavior relationship, an increasing body of evidence shows the differential impact of the environment on EBRB with respect to gender [54-58], age [59], socioeconomic status [59,60] and ethnicity [61,62].

#### Personality

Few studies have combined the personality construct with environmental factors in the prediction of EBRBs. However, consistent evidence shows that the personality factor 'extraversion' moderates the intention – behavior relationship, with more extraverted individuals displaying more consistency [63,64]. This result, which is particularly profound in the physical activity domain, suggests that less extraverted individuals may withdraw from opportunities or they may lack social environments necessary for intention translation [65]. A recent study among adolescents suggested a potential moderating role of the personality factors 'agreeableness' and 'openness to experience' in fruit and vegetable consumption [66].

#### Awareness

Awareness of personal behavior status is likely to influence the level of cognitive energy that is put into intentions to change current EBRBs [67-69]. For example, if a person is not accurately aware of personal physical activity levels, this might lead to a false positive conclusion regarding the compliance with recommended levels. Consequently, unaware individuals lack a sense of urgency, which is hypothesized to lead to an attentional bias regarding relevant environmental cues.

#### Involvement

Involvement can be viewed as the most important feature of the concept of motivation (see e.g., [14]), and refers to "... the complexity or extensiveness of cognitive and behavioral processes characterizing the overall... decision process" ([70] p. 185). If individuals are not involved in a particular behavior, they tend not to put much energy in the decision process [71]. Absence of conscious reflections on behavior is likely to make an individual susceptible to environmental influences, leading to 'spontaneous' execution of behavior [72].

#### Habit strength

Many EBRBs, such as playing outside after school or watching TV for children or taking the bicycle to work for adults, are typically routine behaviors. They are repeatedly performed and may thus be largely determined by habit. Consequently, the concept of habit strength is important in studying these behaviors [73]. Studies have shown that when certain behavior becomes a strong habit, it may follow automatically upon encountering the relevant environmental cues [17]. Thus, the degree of automaticity of a particular environment – behavior relationship will strongly depend on the stability of the environmental cue and on the habitual level of the behavior in question.

#### Engagement in clustered behavior

Studies have shown correlations between physical activity and a prudent diet [74], dietary fat [75], fiber and sucrose intake [76] and fruit and vegetable consumption [77,78], with active individuals having healthier diets. In a sample of adolescents, fruit consumption was found to be positively associated with physical activity during leisure time, and snacking behavior was positively related to using high-fat sandwich fillings [79]. Epidemiologists label the co-occurrence of behaviors as 'clustering' if a combination of behaviors is more prevalent than can be expected on the basis of the prevalence of the separate behaviors [78]. Clustering of behaviors within the energy balance provides evidence for the surplus value of studying multiple clustered behaviors rather than studying behaviors in isolation. For example, EBRBs can be executed simultaneously (e.g., consuming potato chips while watching TV). When specific acts are clustered, they may be simultaneously influenced by identical environmental factors.

## Discussion

Some authors have proposed models that aim to clarify causal relationships between environmental factors and physical activity behaviors (e.g., [80-83]). Similarly, models have been developed for environmental influences on dietary behaviors (e.g., [84]). These models differ substantially on proposed causal mechanisms, specific behavioral acts and target groups. Interestingly, some of these models include notions of dual-processes (e.g., [82-84]), though often not stated explicitly. However, no postulations are made regarding circumstances under which direct or mediated environmental influences will take place. Moreover, these models lack the energy balance-approach, i.e. a focus on both dietary and physical activity behaviors. In previous papers, we have argued that applying such an approach will have multiple benefits, both for determinant studies [23] and intervention programs [85].

Critics (we recommend [86] in this respect) would argue the uselessness of applying a dual-process view, because of a lack of definite proof of its usefulness in the study of determinants or the design of interventions in the field of obesity prevention. In addition, the proposed framework is so broadly defined that it does not generate clear hypotheses regarding specific influential environmental factors. Although we have indications from studies within the NHF-NRG project [85] that the framework does provide an illuminative view on environment – behavior processes and on the development and evaluation of interventions aimed at the prevention of weight gain, future studies are clearly needed to show its usefulness. The EnRG framework, in fact, is the result of an inductive reasoning process. In contrast, deductive reasoning (i.e., moving from a general model to specific observations) is now needed to narrow down the framework to specific testable hypotheses. Thus, the most desirable goal is not the validation of EnRG in its present form but in the evolution of a paradigm that may help to disentangle the role of 'obesogenic' environmental factors (see also [87]).

Rather than focusing on *which *factors may be of importance, the EnRG framework is specifically directed at generating questions related to *when*, *how *and *for whom *environmental factors may be influential. To illustrate this, we will present a set of research questions that can be derived from the EnRG framework. For example:

* How do prompts influence stair use?

Interventions to promote stair use have frequently and successfully applied the strategy of 'prompting', for example using posters with short messages (see [88] for a review). Prompts appear to bring existing beliefs into consciousness, without requiring substantial levels of attention or intention. Prompts can be presented with or without providing a reason ('take the stairs', versus 'taking the stairs is healthy'). Some studies have shown that providing prompts regarding strong beliefs (e.g., 'taking the stairs is healthy') are more effective than those referring to weaker beliefs (e.g., 'taking the stairs saves electricity') [89], indicating the involvement of conscious processing of arguments. However, other studies found no differences between the effectiveness of strong and weak beliefs [90] and a study on environmentally destructive behaviour (walking on a lawn) showed that mere announcement of reasons to act had no effect beyond that already induced by a response-specific prompt [91]. The latter result supports the notion that prompts serve as a peripheral cue rather than as a cue that involves central, conscious, information processing [15]. Insights into these working mechanisms will help intervention designers to optimize their interventions, and to select appropriate target groups and settings.

Other relevant examples of research questions that can be derived from the EnRG framework are:

* How do parental home rules regarding TV- and computer-use determine the screen-viewing behavior of their children?

* For whom are price reductions of fruits at the worksite influential in changing total fruit intake?

* Under which circumstances does the availability of sidewalks influence walking behavior of elderly?

The EnRG framework guides the formulation of specific hypotheses regarding the impact of moderators of environmental influences. For example:

* Parental home rules regarding screen-viewing will have direct impact on adolescents that score high on the personality dimension 'Agreeableness', while the impact will be mediated by social cognitions in adolescents scoring low on this personality characteristic.

* Information on the number of calories in a snack will lead to cognitive processing in those individuals that are highly involved in dietary behavior, while peripheral cues (e.g., color, smell, position on shelf) will have a direct impact on behavior in individuals that are uninvolved.

* The availability of fruit at home will have a direct relation to fruit consumption when this behavior is habitual, but a cognitively mediated impact when fruit consumption behavior is not habitual.

* The impact of the availability of snacks in a school will depend on the price of these snacks.

The latter example is provided in order to illustrate the postulate that types and levels of environments do not operate in isolation, but they are likely to interact. Although empirical evidence regarding such interactions in the field of EBRBs is scarce, studies in the field of child development have shown that the impact of micro-level factors on individual behavioral developmental variability can vary as a function of contextual macro-level conditions [92]. The existence of such 'higher order moderation' has also been suggested in the field of EBRBs. For example, the impact of behavior-specific parenting practices has been hypothesized to be moderated by general parenting styles [93]. A major challenge for future empirical applications of the EnRG framework will be to document under what conditions higher order environmental moderation is most or least likely to occur (see also [94]).

We advocate the application of methodological triangulation [95] in the operationalization of the proposed dual-process view. Multiple types of research are needed, as well as the application of both objective and subjective measures of environmental and behavioral factors. Since environmental factors are postulated to be capable of influencing behavior without individuals being consciously aware of them, researchers cannot only rely on subjective measures of the environment. On the other hand, the individual perception of the nature of the environment, rather than the actual environment, will be critical in determining the mediated route of environmental influences on behavior. In addition, we advocate the use of multiple research designs. Although the EnRG framework is proposed to guide formulation of research questions in specific studies, no single analysis can fully apply or test it. It is the integration of results from cross-sectional studies, cohort studies, large-scale field interventions and small-scale laboratory experiments that is needed to answer hypotheses derived from the framework.

## Conclusion

Studies on the impact of the obesogenic environment have often used non-theoretical approaches. Contributions to the Theory Debate of this journal called for formulations of theories with factors that are unmediated by social cognitions, with increased emphasis on environmental determinants and a focus on potential moderators. These calls fit well within the dual-process view that is incorporated in the EnRG framework. In addition, EnRG applies an energy balance-approach, stimulating the integrated study of determinants of diet and physical activity. The framework may guide research towards causal mechanisms linking specific environmental features with EBRBs in distinct populations. Furthermore, EnRG can inform the design of interventions as well as the formulation of evaluation protocols. Notably, the framework requires the assessment of cognitive mediators and potential moderators in order to illuminate the causality of intervention effects.

## Competing interests

The author(s) declare that they have no competing interests.

## Authors' contributions

SK initiated this paper and wrote the first draft. GJdB, TV, WvM, NdV and JB discussed the draft paper with SK and provided written comments.
